# SentiUrdu-1M: A large-scale tweet dataset for Urdu text sentiment analysis using weakly supervised learning

**DOI:** 10.1371/journal.pone.0290779

**Published:** 2023-08-30

**Authors:** Abdul Ghafoor, Ali Shariq Imran, Sher Muhammad Daudpota, Zenun Kastrati, Sarang Shaikh, Rakhi Batra

**Affiliations:** 1 Dept. of Computer Science, Sukkur IBA University, Sukkur, Pakistan; 2 Dept of Computer Science (IDI), Norwegian University of Science and Technology (NTNU), Gjøvik, Norway; 3 Department of Informatics, Linnaeus University, Växjö, Sweden; Centro de Investigacion en Ciencias de Informacion Geoespacial AC (Research Center on Geospatial Information Sciences), MEXICO

## Abstract

Low-resource languages are gaining much-needed attention with the advent of deep learning models and pre-trained word embedding. Though spoken by more than 230 million people worldwide, Urdu is one such low-resource language that has recently gained popularity online and is attracting a lot of attention and support from the research community. One challenge faced by such resource-constrained languages is the scarcity of publicly available large-scale datasets for conducting any meaningful study. In this paper, we address this challenge by collecting the first-ever large-scale Urdu Tweet Dataset for sentiment analysis and emotion recognition. The dataset consists of a staggering number of 1, 140, 821 tweets in the Urdu language. Obviously, manual labeling of such a large number of tweets would have been tedious, error-prone, and humanly impossible; therefore, the paper also proposes a weakly supervised approach to label tweets automatically. Emoticons used within the tweets, in addition to SentiWordNet, are utilized to propose a weakly supervised labeling approach to categorize extracted tweets into positive, negative, and neutral categories. Baseline deep learning models are implemented to compute the accuracy of three labeling approaches, i.e., VADER, TextBlob, and our proposed weakly supervised approach. Unlike the weakly supervised labeling approach, the VADER and TextBlob put most tweets as neutral and show a high correlation between the two. This is largely attributed to the fact that these models do not consider emoticons for assigning polarity.

## 1. Introduction

We are living in an era where our trust in technology is so strong that the majority of our decisions are influenced by it. Imagine you want to upgrade your phone to its latest released version; it is almost certain that your first step would be to read customers’ reviews about it through online product reviews. From the product owner’s perspective, processing these reviews manually is a nightmare. Therefore in the field of natural language processing, a whole new area of sentiment analysis is assisting in labeling reviews automatically in distinct categories, mostly negative, positive, and neutral. The literature reports two main approaches for processing text for sentiment analysis. The first is lexicon-based approach [1–8] which counts number of positive and negative words to label the text segment whereas the second approach is machine learning [9–13] which exploits different supervised and unsupervised algorithms to extract sentiment from the text.

The Sapir-Whorf Hypothesis [14] states that there are certain thoughts of an individual in one language that cannot be understood by those who live in another language. The hypothesis states that the way people think is strongly affected by their native languages. Therefore, expressing sentiments or opinions is much easier in the mother tongue than in any other language. Expressing feelings of hatred or love is difficult in a second language, whereas seamless in the mother tongue. For example, people from Pakistan would express their feeling or sentiments more freely and realistically while writing in Urdu, whereas Indians would love expressing emotions in Hindi. The field of sentiment analysis has made significant progress in processing English or other resource-rich language text. However, in resource-poor languages like Urdu, the performance of sentiment analysis is still in its infancy. Recently, few attempts have been made to extract sentiment from different languages including Thai [15], Korean [16], Arabic [17], Chinese [18], Portuguese [19] and Malay [20].

According to 2021 estimates of Ethnologue, Urdu is ranked as 10^th^ most widely spoken language of the world, having 230 million speakers (https://www.ethnologue.com/guides/ethnologue200). It is the official language of Pakistan and few parts of India. It is also spoken in many parts of Bangladesh, Nepal, and the Middle East, and a significant diaspora of these countries in Europe, Canada, and the USA. Indeed, it is an understatement to suggest only 230 million speakers of Urdu; a similar number of the population speaks Urdu as their second language. More importantly, Hindi has more than 490 million speakers, and the most dominant language of highly populated India resembles quite significantly with Urdu. Although the two languages’ alphabet is different, from a spoken perspective, both are very similar. Mostly, those who can understand Hindi also understand Urdu and do the conversation without much difficulty.

Urdu is also being extensively used as an internet language with growing news platforms, including BBC-Urdu, Dawn, Express Tribune, and other giant media houses with dedicated Urdu news websites. Social media platforms have also seen a significant rise in the usage of the Urdu language as a communication medium [21].

Despite such a vast population and the importance of the Urdu language, from a machine learning perspective, the Urdu language is still considered a resource-poor language, for it does not have many big datasets, unlike English, Spanish, and other resource-rich languages. For example, there is no equivalent of Sentiment140 [22] in the Urdu language. Most of the recent attempts have, at most, resulted in only a few thousand instances in different datasets. Therefore, natural language processing tasks for the Urdu language, such as classification, summarization, seq2seq modeling, text generation, etc., are still in the infancy phase. The main reason behind the lack of big datasets in the Urdu language, especially from a sentiment analysis perspective, is the absence of exploiting automatic labeling techniques. Most of the datasets available in the Urdu language have been tagged through a manual labeling process, resulting in only a few thousands of instances. SentiUrdu-1M, proposed in this paper, is a large-scale Urdu tweets dataset labeled through innovative, weakly supervised automated techniques. The specific contributions of this work are listed below:

1. Collected a large-scale Urdu tweet dataset called SentiUrdu-1M for sentiment analysis and emotion recognition tasks.
2. Proposed a weakly supervised technique to label the tweets into positive, negative, and neutral polarity. The emoticons along with SentiWordNet, are used to train a model on a subset of the dataset for semi-supervised classification.
3. Established the baseline results on deep learning models on the newly collected large-scale tweet dataset.
4. Compared and evaluated the baseline model results on labeled data obtained via VADER and TextBlob to weakly supervised technique.

We strongly believe that SentiUrdu-1M would cause an advancement in processing Urdu language from an NLP perspective, and the tasks such as text summarization, classification, seq2seq modeling, and Urdu text generation would benefit from it.

The rest of the article is structured as follows. Section 2 presents the related work. A large-scale Urdu tweet dataset is explained in Section 3. Section 4 describes the data annotation and labeling techniques. Experimental settings are provided in Section 5 followed by results and their analysis presented in Section 6. Finally, the conclusion is drawn in Section 7.

## 2. Related work

Sentiment analysis is the study of people’s opinions, attitudes, and emotions toward individuals, businesses, and topics. For example, businesses want to find customers’ views about their products or services, and customers also read other people’s reviews about the product before buying. Sentiment analysis and emotion detection are often used in the same way but are quite different. Emotion is a complex psychological state, such as fear, anger, or happiness. The sentiment is a mental attitude produced by negative, positive, and neutral feelings. To extract sentiment from text, it is necessary to know subjectivity and emotion—two crucial concepts of sentiment analysis.

Subjectivity: subjective sentences comprise personal feelings or beliefs, e.g., opinions, allegations, suspicions, and desires. The subjective sentence may not contain the opinion. For example: “I want a phone with good voice quality” [23]. The sentence seems positive but it is not expressing any opinion.

Emotions: emotions are subjective feelings and thoughts. There are six primary emotions: joy, sadness, fear, anger, surprise, and disgust [24]. Emotions play vital roles in the existence or the complete make-up of individuals. (1) Joy is a pleasant emotional state defined by feelings of happiness, satisfaction, well-being, and gratification, such as smiling, a pleasant way of talking, and a relaxed body language stance. (2) Sadness is defined by feelings of disappointment, sorrow, uselessness, dull mood, crying, quietness, and feeling down are a few ways to express sadness. (3) fear is an emotional state often expressed as a result of perceived danger (4) Anger emotion can be defined by feelings of frustration or hostility towards others. It can be expressed by glaring, turning away, yelling, hitting, or throwing objects. (5) Surprise is another primary emotion that can be defined as a feeling of physiological startling response following something unexpected and expressed by screaming, jumping back, widening the eyes, and opening the mouth. (6) Disgust emotion often results from an unpleasant event that can be expressed by wrinkling the nose and curling the upper lip.

Sentiment analysis on English text is almost a decade and half old, popular works like IMDB dataset [25], Sentiment140 [22], Twitter US Airline Sentiment [26], Amazon Product Reviews [27] etc., has brought the performance of this field at an almost a human level accuracy. However, resource-poor languages still lack decent size (in excess of 100, 000 instances) dataset availability, thus suffering from low performance.

### 2.1 Sentiment analysis for the Urdu language

Recently, many studies have been performed on Urdu text for sentiment analysis. There is a common issue in all these studies, the dataset is limited to a few thousand instances only, and modern deep learning algorithms, which have outperformed traditional machine learning algorithms, are data-hungry. The researchers have proposed several approaches to assign polarity to Urdu text. The majority of researchers have used a manual human-annotated approach for this task; however, few studies have also used multi-lingual and POS Tagging approaches. This section will discuss the recent studies conducted on Urdu text sentiment analysis dataset development.

As discussed above, most research studies have used the manual labeling approach to create an Urdu dataset. Bilal et al. [28] developed an Urdu dataset consisting of 300 samples for Roman-Urdu opinions, and 150 samples for each negative and positive class. Urdu opinions were extracted from blogs and labeled by human annotators. Text classification was performed using three machine learning algorithms: Naïve Bayes, Decision Tree, and KNN. Experimental results revealed that Naïve Bayes performed better than other algorithms. In [29], the authors have proposed the Urdu-based lexicon for sentiment analysis. The authors scraped more than 26000 Urdu tweets from three Twitter accounts: (1) jang_akhbar, (2) BBCUrdu__, and (3) Dawn_News. They manually created an Urdu lexicon word list of 20,171 unique words from tweets and a POS tag was assigned to each word. The study only considered nouns, adjectives, and adverbs. The final list was reduced to 12,808 words. Afterward, human experts in the Urdu language were asked to label the unique nouns, adjectives, and adverbs as negative, positive, and neutral.

Mukhtar et al. [30] have compared the lexicon-based approach with the machine learning approach for Urdu sentiment analysis. The study reveals that the lexicon-based approach outperformed machine learning. The authors collected 6,025 Urdu sentences from 151 blogs to perform their experiments. Two human experts in the Urdu language were hired to annotate the sentences as negative, positive, and neutral. When there was disagreement between two annotators, a third expert was also hired to resolve the difference. For verification, the inter-annotator agreement is calculated by using Kappa statistic [31]. To create the Urdu lexicon, the positive and negative words were collected from three sources. (https://chaoticity.com/urdusentimentlexicon/), (https://sites.google.com/site/datascienceslab/projects/multilingualsentiment), (http://urdulughat.info/). A total number of 11,739 negative and 9,578 positive words were selected. Lexicon-based method raised the accuracy of the machine learning from 73.88% to 89.03%.

Mehmood et al. [32] proposed a discriminative feature spamming method for Roman Urdu sentiment analysis. The study collected 11,000 Roman Urdu reviews from many blogs and social media sites. They used a multi-annotator approach to label the dataset. The proposed approach improved the performance of standard machine learning algorithms. The research study [33] collected Roman Urdu comments from websites and annotated them manually as negative or positive. The final annotated dataset consisted of 400 positive and 406 negative comments and used three machine learning algorithms for classification, namely, Naive Bayes, Logistic Regression with Stochastic Gradient Descent, and Support Vector Machine. The experiment concluded that SVM, with an accuracy of 87.22%, performed better than other classifiers. The authors in paper [34] have proposed the Markov Chains approach for Urdu sentiment analysis. The proposed method consists of manual and probabilistic steps to label the dataset. Initially, 1,400 Urdu tweets were manually annotated by human experts, further to label the remaining 1,703 tweets. The Markov chains method is used to train the model on labeled data and predict the scores for 1,703 unlabeled samples. If the prediction score was more than 80%, then the tweet assigned predicted polarity else labeled manually. The final dataset comprised 328 positive, 1,604 negative, and 1,171 neutral tweets. Furthermore, the proposed method, machine learning, and lexicon-based approaches were evaluated on test data. Experimental results revealed that the proposed approach outperformed the other approaches.

Few recent studies [35–37] have also used a multi-lingual approach to develop datasets for Urdu sentiment analysis. Mukund et al. [37] have proposed the structural correspondence learning method for Urdu sentiment analysis. The study used the IIIT POS Hindi dataset, which was already in Latin script format. The Hindi dataset has many pure Sanskrit words which need to be replaced by Urdu, this replacement is done using online dictionaries (http://www.urduword.com/), (https://hamariweb.com/), and manual lookup. libSVM algorithm was used for text classification, and the algorithm produced an F-measure of 64.3%. Asghar et al., in their paper [35], have used the multi-lingual approach to develop a lexicon-based dataset for Urdu sentiment analysis. They extracted the adjective from the Urdu text using Urdu POS Tagging, then translated Urdu adjectives into English using a multi-lingual Urdu-to-English dictionary. The SentiWordNet lexicon was used to get a sentiment score for translated English adjectives [38].

Syed et al. in paper [39] have proposed the lexicon-based approach for Urdu Sentiment Analysis. 435 movies and 318 product reviews were collected from different websites. Urdu sentiment lexicon is used to assign the sentiment polarity to reviews. Experimental results show that the model produces 72% accuracy on movie reviews and 78% on product reviews. In the research paper [40], the authors have proposed the Roman Urdu Opinion mining system. Mobile phone reviews were collected from Whatmobile (https://www.whatmobile.com.pk/), and Bing translator was used to translate these reviews into English. SharpNLP is used for POS tagging, and adjectives were selected as opinion words. The adjective lexicon dictionary was developed manually to assign the sentiment polarity to reviews. A summary of the related work is depicted in Table 1.

**Table 1:** Urdu sentiment analysis related work summary.

Article	Year	Number of samples	Labeling approach	Sentiment analysis approach
[39]	2010	753	Lexical corpus	Lexicon base
[37]	2012	NA	Multi-lingual	Machine learning
[40]	2015	1620	Multi-lingual & Manual	Lexicon base
[28]	2016	300	Manual	Machine learning
[29]	2017	12,808 words	Manual	Lexicon base
[30]	2018	6,025 sentences and 21,317 words	Manual	Machine learning & Lexicon base
[32]	2019	11,000	Manual	Machine learning
[33]	2019	806	Manual	Machine learning
[35]	2019	9381 words	Multi-lingual	Lexicon base
[34]	2020	3103	Manual & Probabilistic	Markov Chains

Many researchers have also worked on Roman Urdu sentiment analysis. Roman Urdu uses English language characters, while the original Urdu writing uses Urdu language characters. Hussain et al. [41] used the LSTM model to perform Roman Urdu sentiment analysis. The authors compared the proposed model with Naive Bayes, Random Forest, and Support Vector Machine. Their proposed deep learning model outperformed the machine learning models. In the research paper [32], the authors collected 11000 Roman Urdu reviews and labeled the reviews manually, and proposed a novel term weighting technique, called discriminative feature spamming technique (DFST) for sentiment analysis. Lal et al. [42] collected 9601 Roman Urdu reviews from the web and assign them sentiment polarity as negative and positive. For sentiment analysis, the authors proposed deep learning and machine learning models.

## 3 Urdu tweet dataset

SentiUrdu-1M, proposed in this paper, is the first of its kind, a large-scale dataset of tweets in the Urdu language. It facilitates researchers in the field of NLP to perform tweet analysis and to evaluate the existing models and techniques for their accuracy in processing Urdu language text.

This dataset contains 1, 140, 825 tweets and is publicly available on the Mendeley (https://data.mendeley.com/datasets/rz3xg97rm5/1). These tweets are collected by using Twitter Search API “Tweepy”. It scraps Twitter for tweets based on keywords, hashtags, mentioned users, and dates (up to the last seven days). For our purpose, we have used the following query to extract data from Twitter:

$$\begin{array}{c} lang:ur\;until:[specify-end-date]\;since:[specify-start-date]-filter:links \end{array}$$ (1)

This query fetches the tweets that are posted in the Urdu language between specified dates and do not contain links. The extracted raw data contains 72 columns, which describe tweets, users who have posted them, retweet information, and timestamps. We have retained three columns from these in our dataset that are suitable for our purpose. These are tweet id, tweet text, and tweet create date.

In order to make this dataset suitable for machine learning models we have performed pre-processing to remove unnecessary punctuation, spaces, characters, symbols, and mentioned hashtags and users from the tweet text. This large-scale Urdu dataset can be used to improve the sentiment analysis models for low-resource languages, therefore, we have extracted emojis from the tweet text because emojis represent human natural expression very neatly [43], so we can assume the sentiment of a user from the emojis he/she posted in a tweet. Emojis are extracted from tweet text by using a Python script that searches for the emoji in text from a list of 751 most frequently used emojis identified by [44]. These 751 emojis are further classified into different categories based on their representation. The categories are joy, sadness, fear, surprise, disgust, and anger.

**Fig 1:**
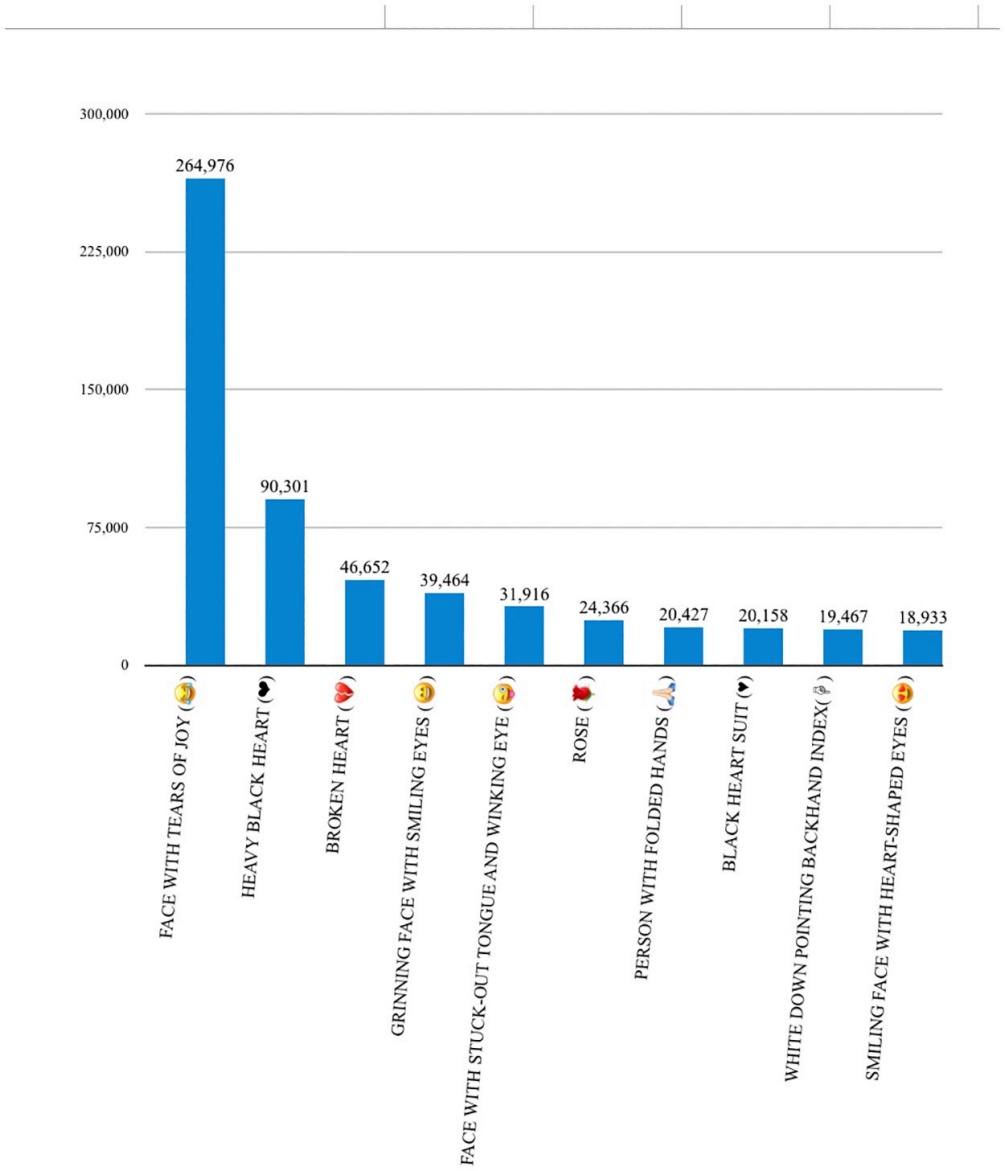
Top 10 frequently used emojis.

In this dataset, it is observed that users have used many different emojis but the emoji “Face with tears of joy” have been used very frequently around 264, 976 times. Fig 1 presents the top 10 most frequently used emojis.

**Fig 2:**
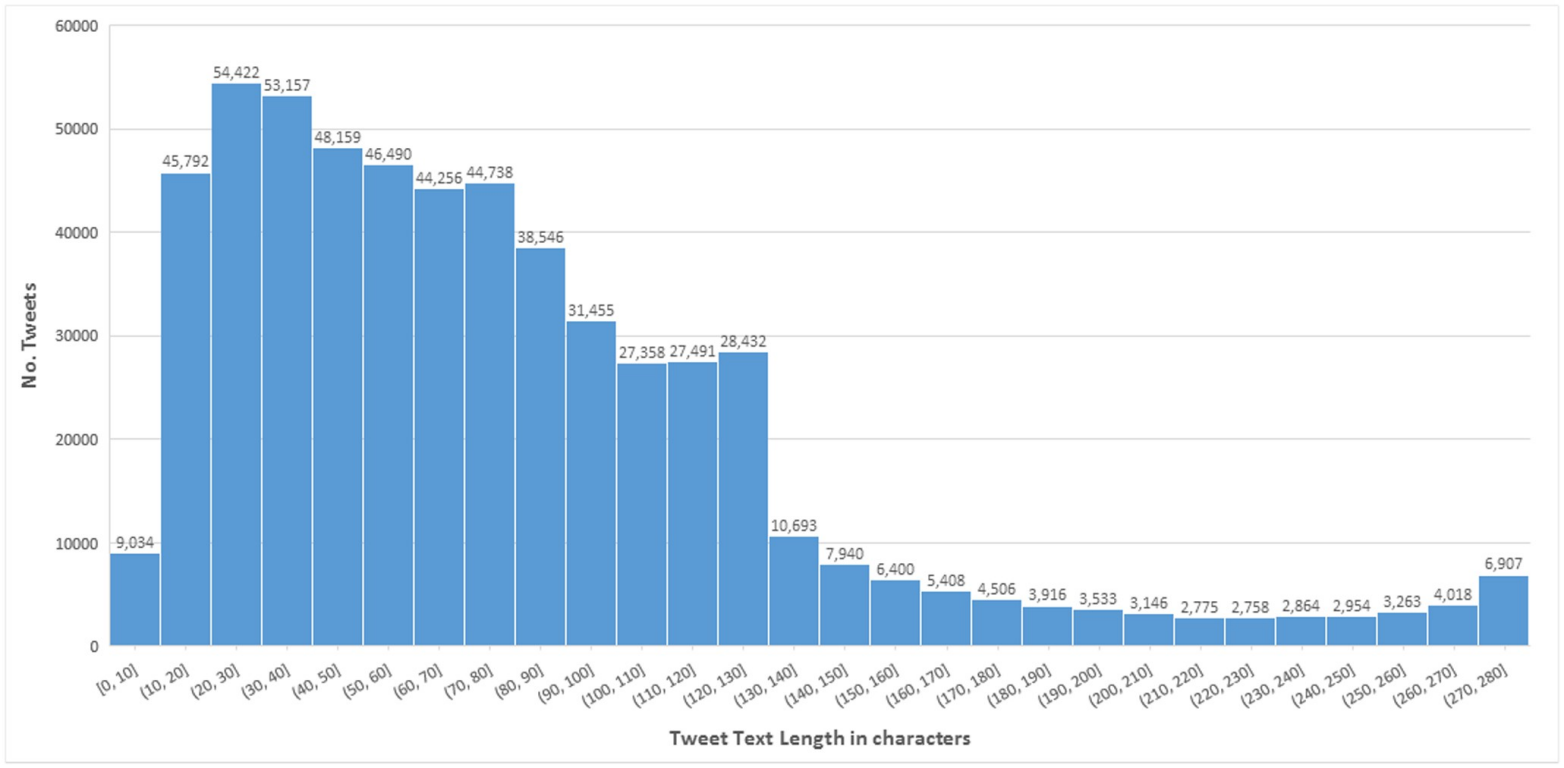
Distribution of tweets on the basis of tweet length.

**Fig 3:**
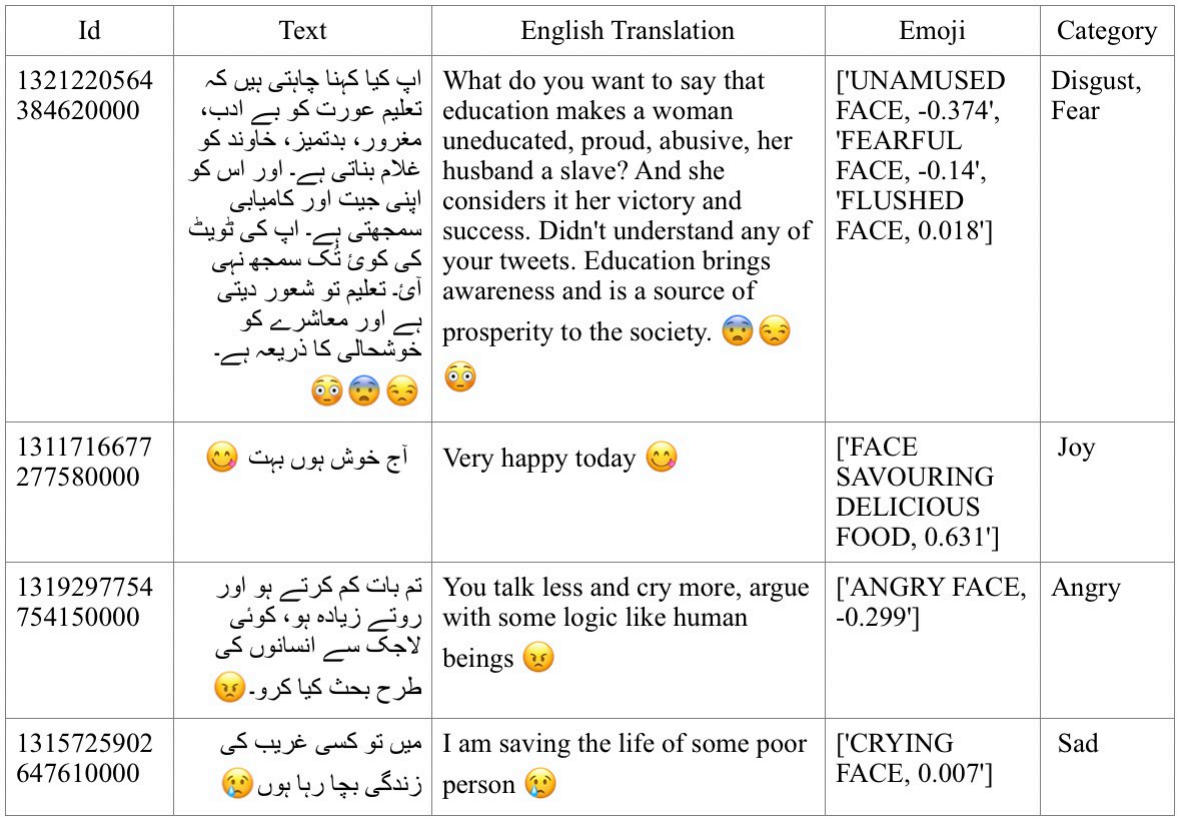
Urdu dataset snippet.

In the final dataset, each tweet record contains tweet id, tweet text, emoji in tweet text, sentiment score of emoji, and category of emoji. Tweet id uniquely identifies each tweet record. Tweet text is the post/content posted by the user, mainly tweet length ranges from 3 to 280 characters. The distribution of the dataset according to tweet length is presented in Fig 2. A dataset snippet is shown in Fig 3.

### 3.1 SentiUrdu1M exploratory data analysis

**Fig 4:**
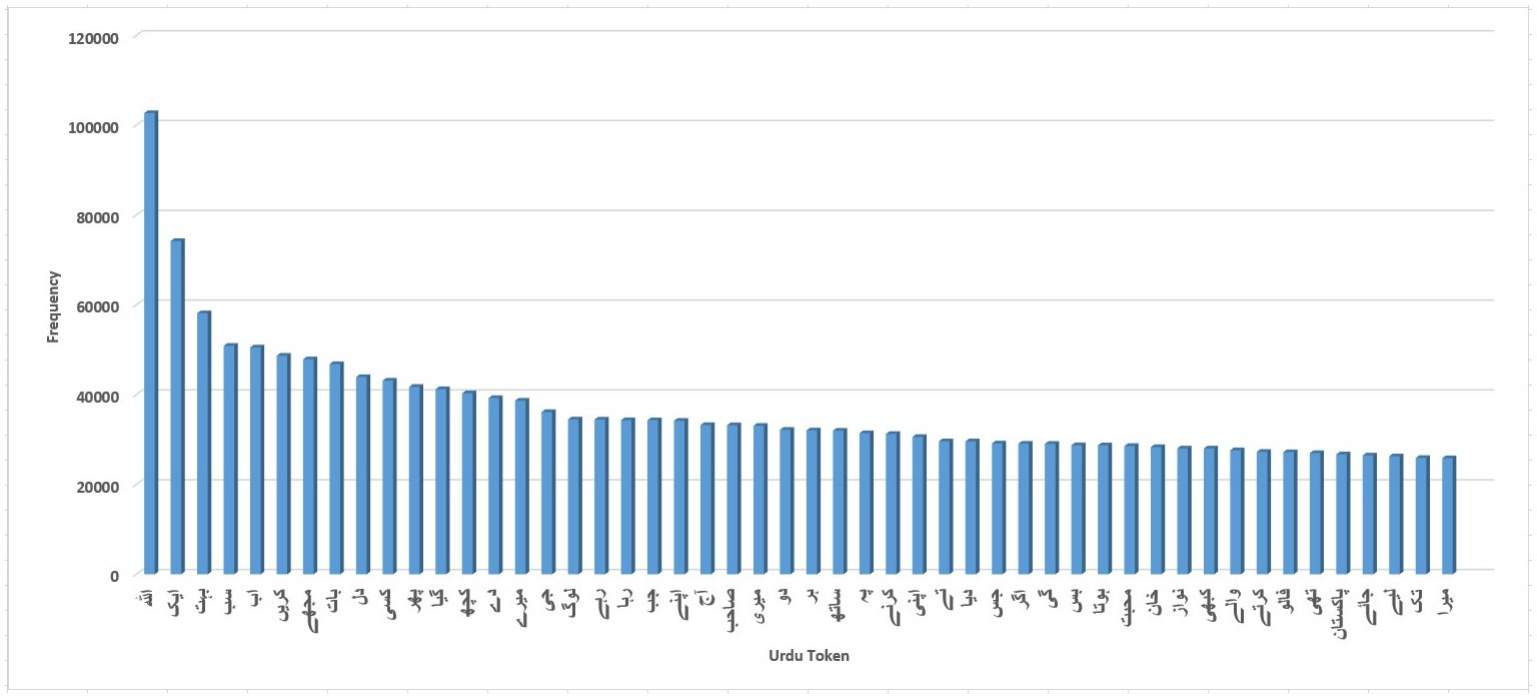
Most frequent words in Urdu dataset.

**Fig 5:**
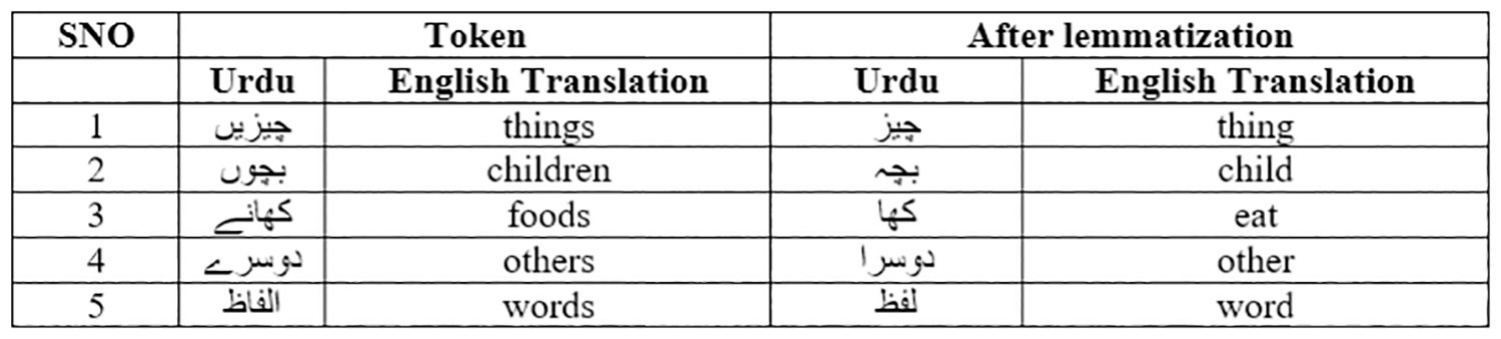
Word inflexions in Urdu.

This study has explored the Urdu text to present essential insights from data. We started to find the most frequent tokens used in the Urdu tweets dataset. Next, we manually read those tokens to extract the 10,000 most frequent Urdu tokens. Some frequent words are depicted in Fig 4, while the complete list can be viewed on the Google drive link (https://drive.google.com/file/d/1FIGdH4ypRSkdrhPOmXBfV-6kf4Czt4yw/view) listing the top 10,000 most frequent tokens publicly for researchers working Urdu language. The shared Google sheet also contains the POS tags and Lemmatization of the top 10,000 Urdu tokens. Further manual analysis was done on the dataset to find whether Urdu contains word inflexions or not. We found many examples of word inflexions by comparing the Urdu tokens and Lemmatization of these tokens, as shown in Fig 5. For example, before Lemmatization, the Urdu word on SNO 1 in Fig 5 means things, but when lemmatizer was applied to the token, words changed to things, and the word “children” changed to a “child”. When lemmatizer is applied to the word “foods,” then the word turns into “eat” which suggests that Lemmatization in Urdu also changes the POS tag of the words (food: noun to eat: verb). This analysis suggests that using original tokens instead of Lemmatization for the Urdu language will be better.

## 4. Methodology

### 4.1 Dataset annotation

Supervised learning algorithms require annotated dataset and the SentiUrdu-1M tweets’ dataset was not initially labeled into sentiment classes: positive, negative, and neutral. It is important to annotate the Dataset into sentiment polarity before it can be used to perform Urdu sentiment classification. There are two main approaches to text data labeling.

1. **Manual Labeling:** This approach requires human experts in the corresponding language to label the text data.
2. **Automatic Labeling:** Programming script is written to automatically label data to avoid manual work.

In order to avoid tedious and error-prone manual labeling, this paper proposes four different auto-labeling approaches, explained below.

### 4.2 Dataset labeling approach 01: Weakly supervised

**Fig 6:**
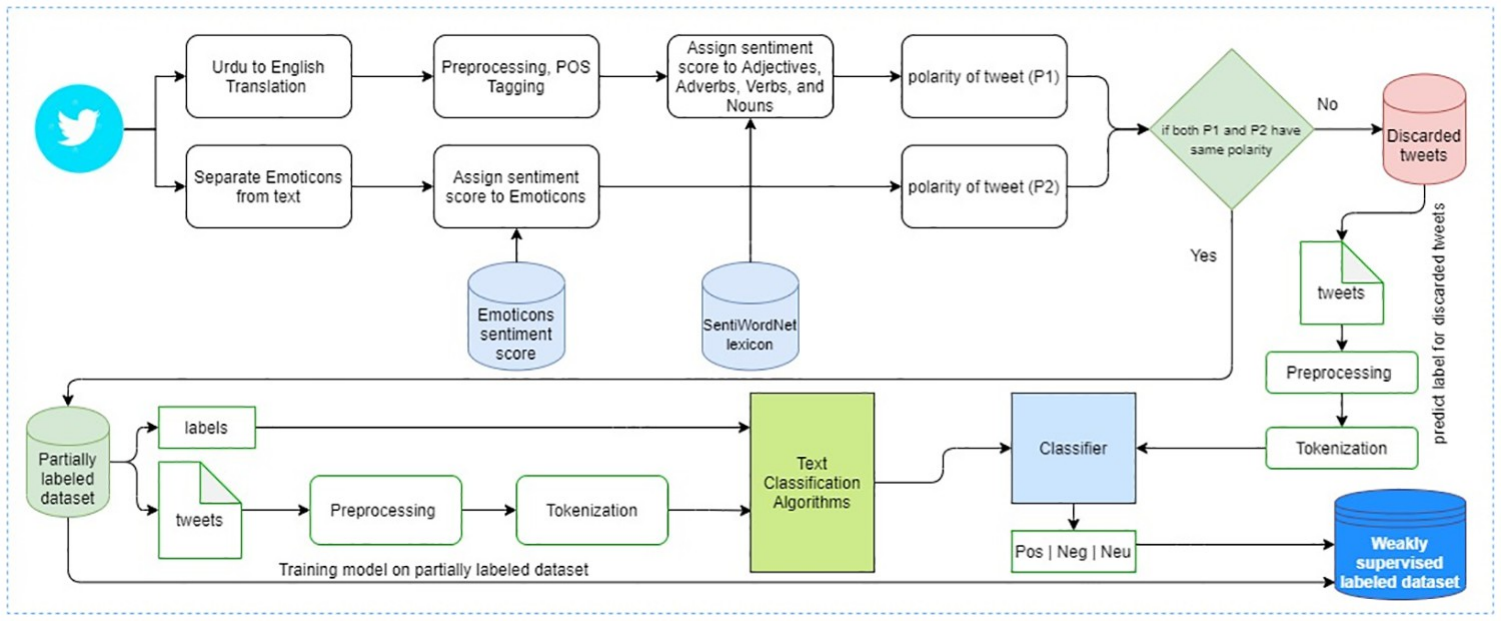
Annotation process of polarity assessment algorithms: Weakly supervised.

The process of auto-labeling tweets’ sentiment polarity through a weakly supervised approach is shown in Fig 6. It considers two inputs for deciding about sentiment polarity. The first input comes from SentiWordnet [38] which is a huge corpus of English language for words’ sentiment polarity score. We start this process by translating Urdu tweets to English using Google Translation API and extracting Adjectives, Adverbs, Verbs, and Nouns from the English text. These words are then queried to SentiWordNet for sentiment scores. Based on the cumulative polarity score, we assign sentiment polarity *P*_1_ either positive, negative, or neutral label.

Similarly, the second input *P*_2_ is based upon emoticon available in Urdu text. We extract emoticons from the text and query the polarity of emoticons from the emoticon sentiment score dataset [44]. The polarity *P*_2_ is either positive, negative, or neutral based upon the emoticon score from the emoticon dataset.

In case both *P*_1_ and *P*_2_ are the same for the input tweet, we retain the tweet in our partially labeled dataset. The reason being we are significantly confident about the polarity of sentiment as two different approaches are voting for the same polarity. In case *P*_1_ and *P*_2_ end up differently, we discard the input tweet at this stage to consider it in the second round of the tagging process. There were 414, 307 tweets out of 1, 140, 823, where sentiment polarities *P*_1_ and *P*_2_ were found the same.

In the second round, we used these 414, 307 tweets and split them into train and test sets, 80% and 20% respectively. Many sentiment classification experiments were performed on these tweets with an 80–20 ratio using deep learning models including LSTM, BiLSTM, and Conv1D. All deep learning algorithms produced almost the same results as shown in Table 2. We train a BiLSTM model on these 414, 307 tweets and use the remaining tweets as a test set on the model for labeling the whole dataset, therefore it was used to predict the polarity of the remaining tweets. Table 3 shows the distribution of the dataset among three labels. The table illustrated in Fig 3 shows a sample of tweets where it can be observed that the emoticons used and the text of the tweet are conveying the same sentiment and emotion polarity.

**Table 2:** Deep algorithms (Partially labeled dataset).

Model	Embeddings	F1-Score					Accuracy
		Positive Class	Negative Class	Neutral Class	Macro Average	Weighted Average	
LSTM [45]	Domain	98.00%	84.00%	78.00%	87.00%	96.00%	96.00%
BiLSTM [46]	Domain	98.00%	84.00%	81.00%	88.00%	96.00%	96.00%
Conv1D [47]	Domain	98.00%	84.00%	83.00%	88.00%	96.00%	96.00%

**Table 3:** Dataset labeling approaches used to assign sentiment polarity to Urdu tweets.

Different polarity assessment algorithms				
	Positive Class	Negative Class	Neutral Class	Total
**Emojis Score + SentiWordNet**	364762	46760	2785	414307
**Weakly supervised**	993144	140036	7641	1140821
**VADER**	6194	2609	1132018	1140821
**TextBlob**	6531	1642	1132648	1140821
**BERT**	531117	448782	160922	1140821

### 4.3 Dataset labeling approach 02: VADER (Valence Aware Dictionary for Sentiment Reasoning)

**Fig 7:**
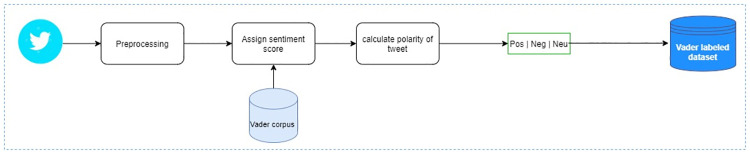
Annotation process of polarity assessment algorithms: Vader.

VADER [48] is a pre-built lexicon as well as a rule-based framework for performing sentiment analysis. It is usually used to assign initial sentiment labeling to the text. A sentiment lexicon is a dictionary where the words are annotated with sentiment scores between -1 and 1. VADER is also able to aggregate sentiment scores of a complete sentence by taking individual scores of words. We used this framework directly from the NLTK package of the Python Programming Language to assign sentiment labels (positive, negative, and neutral) to our Urdu tweets dataset. Fig 7 shows the annotation process of our tweets using this approach. Table 3 shows the individual count of all three sentiment labels assigned using this approach for the tweets.

### 4.4 Dataset labeling approach 03: TextBlob

**Fig 8:**
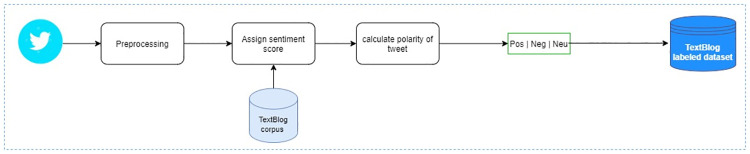
Annotation process of polarity assessment algorithms: TextBlob.

Textblob [49] is a built-in Python library for processing text data. It provides very simple API interfaces to perform various NLP tasks such as part-of-speech tagging, text classification, noun-phrase extraction, and sentiment analysis. For performing sentiment analysis, it uses a sentiment lexicon pattern. to assign sentiment labels (positive, negative, and neutral) to the text. We used this library to assign these three sentiment labels to our Urdu tweets dataset. Fig 8 shows the annotation process of our tweets using this approach. Table 3 shows the individual count of all three sentiment labels assigned using this approach for the tweets.

### 4.5 Dataset labeling approach 04: BERT

This study uses a BERT-based multilingual uncased model(https://huggingface.co/nlptown/bert-base-multilingual-uncased-sentiment) to incorporate an approach from transformer-based models. This version of BERT is fine-tuned for the sentiment analysis on the product reviews dataset. The dataset contains reviews in six languages, namely: English, French, German, Dutch, Italian, and Spanish. Since this model is not trained in Urdu, we first translate it into English using Google translator (https://translate.google.com/) and then provide it as input to the BERT model to predict the sentiment of the text. The results are depicted in Table 3.

## 5 Experimental settings

This section shows different experimental settings we used including deep learning model configurations, training, and test datasets with the relevant evaluation metrics.

### 5.1 Baseline models parameters

Table 4 shows the configuration parameters for all the baseline models we used for the baseline experiments.

**Table 4:** Baseline models configuration parameters.

Sr. #	Model Name	Model Configurations / Parameters
1	DNN [50]	Embedding Layer with 64 Dimension, Dense Layer with 32 Dimension + ReLU, Dense Layer with 3 Dimension + Softmax
2	RNN [51]	Embedding Layer with 64 Dimension, RNN Layer with 32 Dimension, Dense Layer with 3 Dimension + Softmax
3	LSTM [45]	Embedding Layer with 64 Dimension, LSTM Layer with 32 Dimension + Dropout + Recurrent Dropout = 0.2, Dense Layer with 3 Dimension + Softmax
4	BiLSTM [46]	Embedding Layer with 64 Dimension, Bidirectional LSTM Layer with 32 Dimension + Dropout + iw Dropout = 0.2, Dense Layer with 3 Dimension + Softmax
5	Conv1D [47]	Embedding Layer with 64 Dimension, Convolution 1D Layer with 32 Dimension + ReLU + Global Max Pooling 1D Layer, Dense Layer with 3 Dimension + Softmax

### 5.2 Evaluation metrics

**Fig 9:**
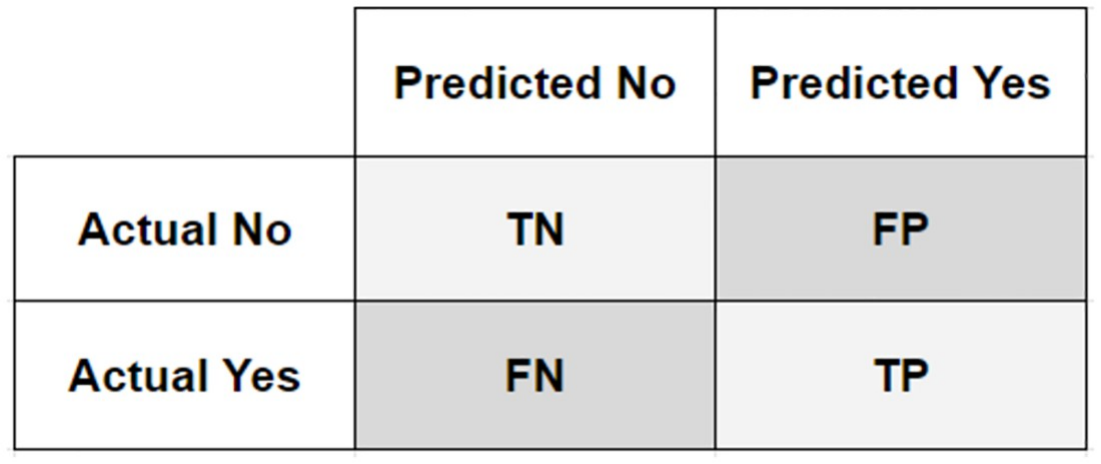
Confusion matrix.

The evaluation metrics are used to evaluate the performance of the system. The most commonly used evaluation metrics for text classification are Precision, Recall, F1-Score, Accuracy, and Kappa Scores. The mathematical representation of all these metrics is given in the Eqs 2, 3, 4, 5 and 6 respectively. All of these metrics are calculated by true positives (TP), false positives (FP), true negatives (TN), and false negatives (FN). These numbers in combination make the confusion matrix as shown in Fig 9.

**Precision**—Precision is the ratio of correctly predicted labels for the specific class in relation to all predicted labels of the class.

$$\begin{array}{c} Precision=\frac{\text{TP}}{\left(\text{TP}+\text{FP}\right)} \end{array}$$ (2)

**Recall**—The recall is the ratio of all predicted labels for the specific class in relation with actual labels of the class.

$$\begin{array}{c} Recall=\frac{\text{TP}}{\left(\text{TP}+\text{FN}\right)} \end{array}$$ (3)

**F1-score**—F1-Score is the harmonic mean / balanced ratio of both the precision and recall.

$$\begin{array}{c} F1-Score=\frac{2\;\times \;(\text{Precision}\;\times \;\text{Recall})}{\left(\text{Precision}+\text{Recall}\right)} \end{array}$$ (4)

**Accuracy**—The ratio of correctly classified labels in relation to actual labels.

$$\begin{array}{c} Accuracy=\frac{\left(\text{TP}+\text{TN}\right)}{\text{TP}+\text{FP}+\text{TN}+\text{FN}} \end{array}$$ (5)

**Kappa Score**—This score expresses the agreement level of an annotation approach versus the ground truth in a classification problem. The returned scores are generally in the range of -1 and 1. Generally, scores greater than 0.81 show perfect agreement.

$$\begin{array}{c} Kappa-Score=(p_{o}-p_{e})/(1-p_{e}) \end{array}$$ (6)

where *p*_*o*_ is the probability value for original class labels and *p*_*e*_ is the probability value for predicated class labels.

### 5.3 Dataset split

Table 2 shows the class-wise as well as overall statistics of the dataset labeled by three different polarity assessment approaches. But in order to carry out the baseline experiments we divided the datasets into three sets. 1) Training Set: 70% of the original dataset, 2) Validation Set: 15% of the original dataset, and 3) Test Set: 15% of the original dataset. The training and validation sets are used by the baseline models to perform training and then we evaluated the performance of those trained models on the test set. Tables 5 and 6 show class-wise as well as total statistics of all of these three sets of datasets labeled by polarity assessment approaches.

**Table 5:** Training + Validation sets of polarity assessment approaches for Urdu tweet dataset.

Polarity Assessment Approach	Different Sets of Datasets used for Baseline Experiments							
	Training Set				Validation Set			
	Positive Class	Negative Class	Neutral Class	Total	Positive Class	Negative Class	Neutral Class	Total
Weakly Supervised	695201	98025	5349	798575	148972	21005	169803	171123
VADER	4336	1826	792413	798575	929	391	169897	171123
TextBlob	4572	1149	792854	798575	980	246	169897	171123
BERT	372040	313995	112542	798575	79476	67315	24332	171123

**Table 6:** Test + Complete sets of polarity assessment approaches for Urdu tweet dataset.

Polarity Assessment Approach	Different Sets of Datasets used for Baseline Experiments							
	Test Set				Complete Set			
	Positive Class	Negative Class	Neutral Class	Total	Positive Class	Negative Class	Neutral Class	Total
Weakly Supervised	148972	21005	1146	171123	993144	140036	7641	1140821
VADER	929	391	169803	171123	6194	2609	1132018	1140821
TextBlob	980	246	169897	171123	6531	1642	1132648	1140821
BERT	79601	67472	24050	171123	531117	448782	160924	1140821

## 6 Results & discussion

This section presents the results of the deep learning models on three polarity assessment approaches discussed in section 3. We also used different conventional machine learning algorithms but the overall results were very poor so we do not report those results. Moreover, the baseline models with domain embeddings performed better than models with general-purpose embeddings i.e. FastText. One possible reason for this could be that FastText is trained on the Wikipedia text. Wikipedia contains formal text mostly written by professionals and Twitter data is a mixture of formal and informal language.

### 6.1 Weakly supervised dataset results

This section shows the results of all the baseline experiments performed on a weakly supervised dataset. The given results are computed against the test set of the dataset. Table 11 shows the precision and recall results and Table 7 depicts the F1-score, accuracy, and kappa score values of individual class labels as well as the whole test set.

**Table 7:** Deep algorithms (Weakly supervised Baseline F1-Score, Kappa Score, and Accuracy).

Model	F1—Score	Kappa Score	Accuracy
DNN	86.00%	81.34%	95.86%
RNN	86.00%	80.58%	95.56%
LSTM	89.00%	84.37%	96.46%
BiLSTM	89.00%	84.67%	96.57%
Conv1D	88.00%	82.37%	96.06%
BiLSTM + FastText	84.00%	16.96%	87.79%

As we can see from Table 3, the dataset is highly imbalanced because a majority of the instances lie in the positive class label so it would be interesting to see either we got satisfactory precision results for low-instances class labels as well. The neutral class has the lowest instances as compared to the positive and negative classes. If we look into the individual or average precision results in Table 11, the top performing baseline models are LSTM, BiLSTM, and Conv1D with 90%+ values. The DNN and RNN models have performed low as compared to other models. The possible reason for the low performance of DNN could be that it is built for processing a single unit of information at a time and is inefficient for processing sequential information such as text sequences. Moreover, the lowest performance of RNN is due to its inability to process and understand long text sequences. The top-performing models have the ability to overcome all of the above-mentioned problems while processing text sequences.

Furthermore, if we observe the individuals as well as average recall values in Table 11, the same models (LSTM, BiLSTM, and Conv1D) are top-performing models. However, the DNN and RNN have low recall values as compared to other models. This gives more confidence regarding the performance of top models (LSTM, BiLSTM, and Conv1D).

Next, we calculated the F1-score, accuracy, and kappa scores of the baseline experiments for the test set of the Weakly supervised dataset. The results are given in Table 7. Here, we can also observe that the top performing models are again the same (LSTM, BiLSTM, and Conv1D) in F1-score as well as accuracy values with 95%+ values. The main values which need to be discussed here are the kappa score values which show the agreement level between the original and predicted class labels. We can see that the overall score values are above 80% which indicates that the predicated class labels are very much near to the original class labels of the dataset. Hence, it gives more confidence in the validity of the baseline experiment results.

### 6.2 VADER dataset results

This section explains the results of all the baseline experiments performed on the VADER dataset. Table 12 depicts the precision as well as recall results. However, Table 8 shows the F1-score, accuracy, and kappa score results of the dataset. The difference in this dataset as compared to the previous one is that the dataset has the majority of the instances in the neutral class. So, it will be exciting to analyze the overall results as well as the results of classes having fewer instances, i.e. positive and negative. The results are computed on the test set of the dataset. The precision results for the neutral class are 100% due to having the majority of the instances. Although the positive and negative classes are with fewer instances, the top-performing models (LSTM, BiLSTM, and Conv1D) have given 90%+ and 80%+ precision results for positive and negative classes, respectively. Moreover, the overall precision and recall results are above 90% and 80%, respectively for the (LSTM, BiLSTM, and Conv1D) models. Again, here the low-performance models are DNN and RNN.

**Table 8:** Deep learning algorithms (VADER Baseline F1-Score, Kappa Score, and Accuracy).

Model	F1—Score	Kappa Score	Accuracy
DNN	83.00%	79.56%	99.72%
RNN	78.00%	75.73%	99.66%
LSTM	87.00%	83.92%	99.76%
BiLSTM	89.00%	86.23%	99.79%
Conv1D	86.00%	81.89%	99.75%
BiLSTM + FastText	88.00%	73.49%	99.64%

Furthermore, the results from the Table 8 give strong evidence for top-performing models (LSTM, BiLSTM, Conv1D) because the F1-score and accuracy values are again taking lead for these models as compared to DNN and RNN models. The kappa score values are interesting to see as again the relation of original to predicted class labels is very strong. The shifting of the majority of the instances from positive to neutral class in this dataset has not affected the overall model’s performance and results.

### 6.3 TextBlob dataset results

This section shows the results of all the baseline experiments performed on the TextBlob dataset. Table 13 shows the precision, and recall results, and Table 9 shows the F1-score, accuracy, and kappa score results of the dataset. Again, this dataset has the majority of the instances in the neutral class. The results are computed on the test set of the dataset. As discussed in the previous section, it is always best to see the performance of the models for classes having low instances as compared to the majority instances classes. Continuing the pattern of previous baseline experiments, again the (LSTM, BiLSTM, and Conv1D) models are taking the lead in individual classes as well as whole test set precision and recall results by giving 90%+ and 80%+ values, respectively. The low-performing models are DNN and RNN. Table 9 also depicts the same top-performing and low-performing models using F1-scores, accuracy, and kappa score values. Here, the dataset has again the majority of the instances in neutral class and it did not affect the overall model’s performance.

**Table 9:** Deep learning algorithms (TextBlob Baseline F1-Score, Kappa Score, and Accuracy).

Model	F1—Score	Kappa Score	Accuracy
DNN	84.00%	85.37%	99.81%
RNN	78.00%	75.31%	99.69%
LSTM	90.00%	89.07%	99.84%
BiLSTM	90.00%	89.39%	99.85%
Conv1D	91.00%	89.85%	99.86%
BiLSTM + FastText	81.00%	79.28%	99.74%

### 6.4 BERT dataset results

Compared to other approaches, BERT results are poor, as presented in Table 10. The Kappa score results suggest that the worst agreement for annotations and the F-1 score, and the accuracy are significantly less than our proposed weakly supervised method. The possible reason is that the Bert-base multilingual uncased sentiment model is fine-tuned on a wide range of product reviews written in six different languages: English, Dutch, German, French, Spanish, and Italian (https://huggingface.co/nlptown/bert-base-multilingual-uncased-sentiment). In our study, we aimed to tackle the challenge of sentiment analysis for content in the Urdu language. To address this, we initially translated Urdu tweets into English and then used the Bert-based model to predict the sentiment of the Urdu content.

**Table 10:** Deep learning algorithms (BERT Baseline F1-Score, Kappa Score, and Accuracy).

Model	F1—Score	Kappa Score	Accuracy
DNN	62.00%	11.00%	68.00%
RNN	62.00%	10.00%	68.00%
LSTM	64.00%	14.00%	70.00%
BiLSTM	64.00%	12.00%	70.00%
Conv1D	64.00%	12.00%	70.00%

Our research findings show that simply fine-tuning a Bert-based model on languages with rich linguistic resources does not necessarily lead to improved performance on languages with fewer resources. This is the case even if we try to bridge the gap by translating resource-poor data into a language with richer resources. We previously explored this issue in detail in one of our earlier research papers [52]. In contrast to the BERT, the proposed weakly supervised method also includes emoticons for identifying the sentiment polarity of Urdu tweets, not taken into consideration by other models.

### 6.5 Comparison of classification results between labelling approaches

**Fig 10:**
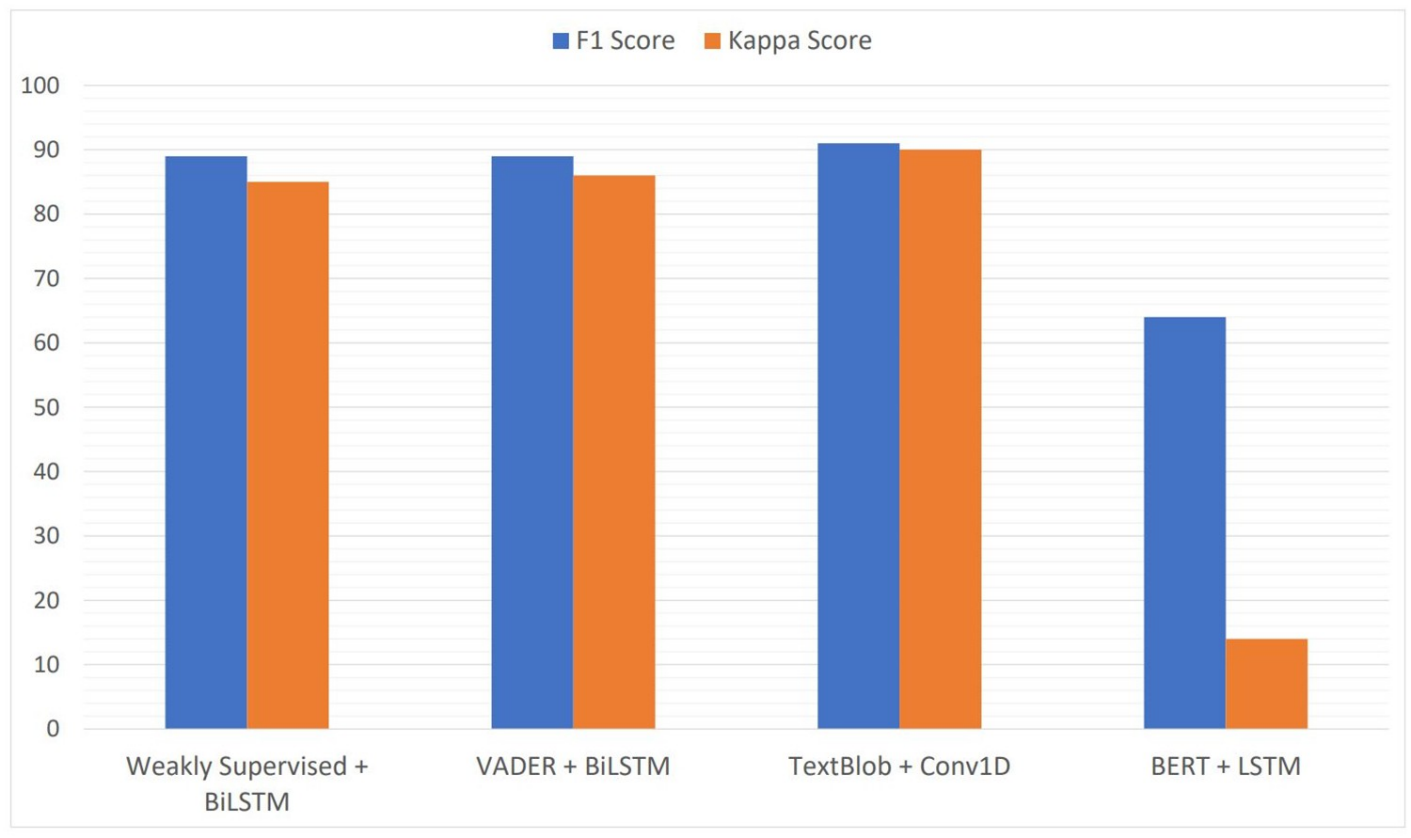
Comparison of classification results between labeling approaches.

This section discusses the comparison of F1-score and kappa score values for best-performing models of all four polarity assessment approaches. The reason to choose F1-score and kappa score is that the F1-score values are the balanced values representing both precision and recall values. Also, the kappa score values are the relation of predicted class labels to original class labels. Fig 10 shows the comparison discussed above.

From Fig 10 we can observe that the best models for all four polarity assessment approaches (weakly supervised, VADER, TextBlob, and BERT) with respect to F1-score and kappa score values are LSTM, BiLSTM, and Conv1D. Also, there is very little difference in F1-score and kappa score values in for weakly Supervised, VADER, and Texblob. This gives strong evidence that whatever polarity assessment approach we use to assign polarities, it will not affect the overall performance and learning of the deep learning models.

The VADER and TextBlob put most tweets as neutral. This is primarily attributed to the fact that these models do not consider emoticons for assigning polarity, which is the main disadvantage of VADER and TextBlob. Still, a high correlation was observed between the two approaches.

Texblob also uses Google Translate to translate low-resource languages such as Urdu into English and generate a polarity class for input text (https://thinkinfi.com/natural-language-processing-using-textblob/), (https://thinkinfi.com/natural-language-processing-using-textblob/). Our recent study conducted in [52] proved that Google Translate caused performance degradation for low-resource languages. Therefore, we did not report extended results in this study to prove it. For more detailed information on this topic, the readers are advised to refer to this research work [52]. TextBlob lexicon solely considers the text to assign polarity. It does not consider the emoticons, so effectively detecting sarcasm, negation, ambiguous words, phrase and idioms, and slang in low-resource languages is often challenging. These terms are very important and can cause polarity changes for the low-resource text. The same case is with VADER. VADER is an English rule-based lexicon that uses the machine translation tool “My Memory Translation Service” (http://mymemory.translated.net) (http://mymemory.translated.net) to generate the polarity for non-English text (https://github.com/cjhutto/vaderSentiment) (https://github.com/cjhutto/vaderSentiment).

The detailed results of the different labeling approaches can be found in Tables 11–14. When compared to other methods, our proposed weakly supervised learning approach stands out for being more balanced and fair. This is evident from the precision and recall values for each class in Table 11. Particularly, the BilSTM model shows the best performance among all models, managing to achieve a well-rounded performance across different classes and an overall average of 91%.

**Table 11:** Deep learning models (Weakly Supervised Baseline Precision and Recall).

Model	Embeddings	Precision				Recall			
		Positive Class	Negative Class	Neutral Class	Macro Average	Positive Class	Negative Class	Neutral Class	Macro Average
DNN	Domain	97.00%	86.00%	79.00%	88.00%	98.00%	81.00%	76.00%	85.00%
RNN	Domain	98.00%	83.00%	73.00%	85.00%	97.00%	83.00%	80.00%	87.00%
LSTM	Domain	98.00%	87.00%	84.00%	90.00%	98.00%	86.00%	80.00%	88.00%
BiLSTM	Domain	98.00%	88.00%	86.00%	91.00%	98.00%	85.00%	78.00%	87.00%
Conv1D	Domain	97.00%	86.00%	83.00%	89.00%	98.00%	83.00%	78.00%	86.00%

**Table 12:** Deep learning algorithms (VADER Baseline Precision and Recall).

Model	Embeddings	Precision				Recall			
		Positive Class	Negative Class	Neutral Class	Macro Average	Positive Class	Negative Class	Neutral Class	Macro Average
DNN	Domain	91.00%	86.00%	100.00%	93.00%	77.00%	52.00%	100.00%	76.00%
RNN	Domain	83.00%	76.00%	100.00%	86.00%	74.00%	43.00%	100.00%	72.00%
LSTM	Domain	92.00%	83.00%	100.00%	91.00%	82.00%	69.00%	100.00%	84.00%
BiLSTM	Domain	92.00%	83.00%	100.00%	92.00%	86.00%	72.00%	100.00%	86.00%
Conv1D	Domain	96.00%	88.00%	100.00%	94.00%	75.00%	65.00%	100.00%	80.00%

**Table 13:** Deep learning algorithms (TextBlob Baseline Precision and Recall).

Model	Embeddings	Precision				Recall			
		Positive Class	Negative Class	Neutral Class	Macro Average	Positive Class	Negative Class	Neutral Class	Macro Average
DNN	Domain	92.00%	95.00%	100.00%	96.00%	85.00%	49.00%	100.00%	78.00%
RNN	Domain	87.00%	69.00%	100.00%	85.00%	69.00%	50.00%	100.00%	73.00%
LSTM	Domain	92.00%	77.00%	100.00%	90.00%	91.00%	80.00%	100.00%	90.00%
BiLSTM	Domain	94.00%	86.00%	100.00%	93.00%	89.00%	74.00%	100.00%	88.00%
Conv1D	Domain	95.00%	93.00%	100.00%	96.00%	89.00%	72.00%	100.00%	87.00%

**Table 14:** Deep learning algorithms (BERT Baseline Precision and Recall).

Model	Embeddings	Precision				Recall			
		Positive Class	Negative Class	Neutral Class	Macro Average	Positive Class	Negative Class	Neutral Class	Macro Average
DNN	Domain	73.00%	69.00%	44.00%	62.00%	75.00%	69.00%	40.00%	61.00%
RNN	Domain	72.00%	69.00%	46.00%	62.00%	76.00%	70.00%	38.00%	61.00%
LSTM	Domain	76.00%	71.00%	49.00%	65.00%	76.00%	74.00%	42.00%	64.00%
BiLSTM	Domain	74.00%	72.00%	49.00%	65.00%	79.00%	71.00%	40.00%	63.00%
Conv1D	Domain	74.00%	71.00%	49.00%	64.00%	77.00%	71.00%	42.00%	63.00%

Taking a closer look at the outcomes for Vader and TextBlob in Tables 12 and 13, respectively, it’s clear that both approaches tend to favor the Neutral class. This trend is supported by the findings in Table 5, where most Urdu tweets are labeled as Neutral by both Vader and TextBlob. This bias stems from the fact that Vader and TextBlob rely on English language patterns and use translation to handle non-English text. On the other hand, the results from the BERT-based model don’t show a strong bias towards any particular class. However, it’s important to note that the BERT-based model’s performance is noticeably weaker when compared to our proposed weakly supervised method, as highlighted in Table 14.

Table 15 provides a thorough overview of the models that perform the best using various labeling techniques. Among these techniques, TextBlob stands out with better F1 scores and Accuracy. Weakly Supervised and Vader have similar F1 scores, but Vader has higher accuracy. On the other hand, BERT doesn’t perform as well as the other methods.

**Table 15:** Top-performing models for different labeling approaches: A comprehensive overview.

Labeling Approach	Model	F1 Score	Accuracy
Weakly Supervised	BiLSTM	89.00%	96.57%
VADER	BiLSTM	89.00%	99.79%
TextBlob	Conv1D	91.00%	99.86%
BERT Based	LSTM	64.00%	70.00%

It’s important to note that VADER and TextBlob show higher accuracy because they deal with a lot of instances that are categorized as Neutral. This large number of Neutral instances introduces some bias, which leads to inflated accuracy scores for both models. This becomes clearer when we look at Tables 12 and 13, the results for the positive and negative classes aren’t as good when compared to the suggested weakly supervised approach.

### 6.6 Comparison between human labeled and automatic labeled tweets

The dataset proposed in this study contains more than 1 million tweets, so it is impossible to manually annotate this huge dataset. Therefore, in this study, we manually labeled 400 tweets to report human analysis of the dataset, 164 tweets annotated as positive, 158 as negative, and 78 tweets labeled as neutral. Further, we compared manually labeled tweets with automatically labeled methods discussed in this paper, Weakly Supervised, Vader, and TextBlob. This analysis discovered that labeling similarity between human-labeled and proposed weakly-supervised approaches is about 51.5% which is better than the 21.0% and 25.0% respectively for Vader and TextBlob.

## 7. Conclusion and future work

This article aimed to propose a new dataset—SentiUrdu-1M, a large-scale tweet dataset for Urdu language text sentiment analysis. The article also sets baseline results on the SentiUrdu-1M dataset for future researchers to pursue further. Urdu language, despite being spoken by more than 270 million people around the world, is still considered a resource-poor language from a machine learning perspective. Only a handful of datasets with a few thousand instances are available for Urdu text processing, which makes the Urdu language a poor candidate for processing using state-of-the-art deep learning algorithms. SentiUrdu-1M would prove to be a leapfrog in the advancement of the Urdu language and its progress in text processing, especially from a sentiment analysis perspective.

The article also proposed an automated instances-labeling approach using SentiWordNet and emoticons extracted from text. The proposed approach is generalizable and can be exploited to label tweets from other natural languages too as the language of emoticons is universal and corresponding emotions have the same meaning in all human languages. A smiling face is positive in Urdu as well as in Thai, Norwegian, or any other natural language.

SentiUrdu-1M can also be used to train models such as LSTM or GPT-2 for Urdu text generation. Recently, with the advent of attention-based transformer models, the dream of the AI community to generate synthetic text has come true but it is mostly limited to English and a few other resource-rich languages. SentiUrdu-1M has the potential to cause a similar disruption in the Urdu language. This might turn out to be a baby step towards an Urdu-talking robot.

SentiUrdu-1M can also be used to produce a generic pre-trained Urdu word embedding—on similar lines as GloVe-Twitter word embedding for English tweets. Such an embedding would serve a purpose in Urdu text classification, summarization, seq2seq modeling, and other natural language processing tasks.

## Supporting information

S1 Appendix (PDF)
