# Supplementary material for: SentiUrdu-1M: A large-scale tweet dataset for Urdu text sentiment analysis using weakly supervised learning
Source: PLoS One. 2023 Aug 30;18(8):e0290779. doi: 10.1371/journal.pone.0290779 (PMC10468080; doi:10.1371/journal.pone.0290779)
Supplement: S1 Appendix — (PDF) [file pone.0290779.s001.pdf]

S1 Appendix

Table 16. Deep Algorithms (Weakly supervised Baseline F1-Score, Kappa Score, and Accuracy)

Model	Embeddings	F1 - Score					Kappa Score	Accuracy
		Positive Class	Negative Class	Neutral Class	Macro Average	Weighted Average		
DNN	Domain	98.00%	84.00%	77.00%	86.00%	96.00%	81.34%	95.86%
RNN	Domain	97.00%	83.00%	76.00%	86.00%	96.00%	80.58%	95.56%
LSTM	Domain	98.00%	86.00%	82.00%	89.00%	96.00%	84.37%	96.46%
BiLSTM	Domain	98.00%	87.00%	82.00%	89.00%	97.00%	84.67%	96.57%
Conv1D	Domain	98.00%	85.00%	81.00%	88.00%	96.00%	82.37%	96.06%
BiLSTM	FastText	94.00%	12.00%	22.00%	84.00%	43.00%	16.96%	87.79%

Table 17. Deep Learning Algorithms (VADER Baseline F1-Score, Kappa Score, and Accuracy)

Model	Embeddings	F1-Score					Kappa Score	Accuracy
		Positive Class	Negative Class	Neutral Class	Macro Average	Weighted Average		
DNN	Domain	83.00%	65.00%	100.00%	83.00%	100.00%	79.56%	99.72%
RNN	Domain	78.00%	55.00%	100.00%	78.00%	100.00%	75.73%	99.66%
LSTM	Domain	87.00%	75.00%	100.00%	87.00%	100.00%	83.92%	99.76%
BiLSTM	Domain	89.00%	77.00%	100.00%	89.00%	100.00%	86.23%	99.79%
Conv1D	Domain	84.00%	75.00%	100.00%	86.00%	100.00%	81.89%	99.75%
BiLSTM	FastText	75.00%	67.00%	100.00%	88.00%	100.00%	73.49%	99.64%

Table 18. Deep Learning Algorithms (TextBlob Baseline F1-Score, Kappa Score, and Accuracy)

Model	Embeddings	F1-Score					Kappa Score	Accuracy
		Positive Class	Negative Class	Neutral Class	Macro Average	Weighted Average		
DNN	Domain	88.00%	65.00%	100.00%	84.00%	100.00%	85.37%	99.81%
RNN	Domain	77.00%	58.00%	100.00%	78.00%	100.00%	75.31%	99.69%
LSTM	Domain	92.00%	79.00%	100.00%	90.00%	100.00%	89.07%	99.84%
BiLSTM	Domain	91.00%	80.00%	100.00%	90.00%	100.00%	89.39%	99.85%
Conv1D	Domain	92.00%	81.00%	100.00%	91.00%	100.00%	89.85%	99.86%
BiLSTM	FastText	82.00%	62.00%	100.00%	81.00%	100.00%	79.28%	99.74%

Table 19. Deep Learning Algorithms (BERT Baseline F1-Score, Kappa Score, and Accuracy)

Model	Embeddings	F1-Score					Kappa Score	Accuracy
		Positive Class	Negative Class	Neutral Class	Macro Average	Weighted Average		
DNN	Domain	74.00%	69.00%	42.00%	62.00%	68.00%	11.00%	68.00%
RNN	Domain	74.00%	69.00%	42.00%	62.00%	68.00%	10.00%	68.00%
LSTM	Domain	76.00%	72.00%	45.00%	64.00%	70.00%	14.00%	70.00%
BiLSTM	Domain	76.00%	72.00%	44.00%	64.00%	70.00%	12.00%	70.00%
Conv1D	Domain	76.00%	71.00%	45.00%	64.00%	69.00%	12.00%	70.00%
